# Novel Blueberry Leaf Polysaccharide–Xanthan Gum Composite Gels for Curcumin Encapsulation: Enhanced Stability and Controlled Release

**DOI:** 10.3390/foods14162825

**Published:** 2025-08-14

**Authors:** Chuyan Wang, Xiaoying Liu, Yan Zhang, Xiaomin Li, Yuanfei Ge, Wei Lan, Liuqing Yang

**Affiliations:** 1 Anhui Ecological Fermentation Engineering Research Center for Functional Fruit Beverage, Fuyang Normal University, Fuyang 236037, China; 2 College of Biology, Food & Environment, Hefei University, Hefei 230601, China; liuxiaoyingwy@163.com (X.L.); 17856075289@163.com (Y.Z.); xiaominli67@163.com (X.L.); geyuanfei1229@163.com (Y.G.); yangliuqing0928@163.com (L.Y.); 3 Key Laboratory of Berry Processing and Resource Comprehensive Utilization, Hefei University, Hefei 230601, China

**Keywords:** blueberry leaf polysaccharide, composite gel, gel properties, curcumin, protective effect, controlled release

## Abstract

Curcumin is a natural active ingredient with various health benefits but suffers from poor water solubility, chemical instability, and rapid metabolism. This study developed a novel composite gel, blueberry leaf polysaccharide–xanthan gum (BLP-XG), for the protection and delivery of curcumin. The experimental results demonstrate that the formation of stable composite gel networks is predominantly facilitated by hydrogen bonding and electrostatic interactions between BLP and XG components. In comparison with single-component systems, composite gels exhibit superior structural homogeneity and density, as well as higher thermal stability, viscoelasticity, and predominantly elastic solid behavior. The BLP-XG composite gel achieved the highest curcumin encapsulation rate of 84.23% when the BLP concentration was 2.0%. The composite gel system effectively retained curcumin in the gastric juice and released it in the small intestine. Furthermore, the presence of BLP in the composite gel inhibited curcumin degradation under UV irradiation. This study establishes the research foundation for the development of efficient and stable delivery systems to protect and deliver curcumin and extends the use of blueberry leaf polysaccharides in food and pharmaceutical applications.

## 1. Introduction

Curcumin, a natural polyphenolic compound, possesses a variety of functional biological properties, including anti-inflammatory, antibacterial, and antioxidant effects [1]. This substance has been utilized in the treatment of a wide range of diseases, including neurological, cancerous, cardiovascular, pulmonary, metabolic, and liver disorders [2]. However, the industrial application of curcumin as a nutritional supplement or pharmaceutical ingredient is significantly constrained by its poor aqueous solubility and inherent instability. These properties result in curcumin undergoing rapid degradation under conditions of alkaline pH, exposure to light, oxidative stress, and elevated temperatures [3]. In order to enhance the bioavailability of curcumin and improve its oral medicinal effect, a series of delivery systems were utilized, including hydrogel [4], emulsions [5], and colloids [6]. Gel-based delivery systems have been shown to protect curcumin from degradation through effective encapsulation and controlled release. Among them, gel systems formed by polysaccharide–polysaccharide or polysaccharide–protein complexes possess a distinctive three-dimensional network structure, which renders them optimal delivery vehicles for hydrophilic and hydrophobic bioactive ingredients [7]. Plant-derived polysaccharides have unique advantages as environmentally sustainable biomaterials and have become one of the most promising carriers due to their significant hydrophilic properties and excellent rheological properties [8].

Xanthan gum (XG) is an exopolysaccharide generated by the fermentation process of the Gram-negative bacterium Xanthomonas campestris [9] and serves as a vital food thickener and stabilizer [10]. However, due to its poor solubility, inadequate rheological properties, and limited thermal stability, XG does not meet the application requirements of complex food systems [11]. Consequently, researchers have sought to modify XG through chemical and physical crosslinking to address these limitations. Some studies have enhanced the physical, chemical, and rheological properties of XG by adjusting the ratio of carboxymethyl to monochloroacetic acid, while others have achieved chemical crosslinking primarily with citric acid and glycerol as crosslinking agents, resulting in a mixture of XG with improved performance [12]. Conversely, physical crosslinking primarily occurs through the formation of polymers with other polysaccharides or macromolecules, such as proteins, via ionic bonds, hydrogen bonds, and hydrophobic interactions. For instance, Hou et al. synthesized a composite gel using Bletilla striata polysaccharide and XG through hydrogen bonding, which enhanced the viscosity of XG and improved its structural properties [13]. Polysaccharide composite systems have demonstrated significant advantages in terms of both performance and application prospects [14], yet the modulation of XG structure and properties by polysaccharides necessitates further investigation.

Blueberry leaves—an abundant byproduct of cultivation—remain understudied despite their rich composition of bioactive compounds. Notably, BLP constitutes a significant fraction of these bioactive components [15]. Our research group successfully isolated polysaccharides from blueberry leaves and characterized their structural properties, elucidating the primary carbon skeleton configuration [16]. Plant-derived polysaccharides typically exhibit distinctive rheological properties and gelation capacity, attributable to their carboxyl and hydroxyl functional groups that facilitate molecular interactions and enable modulation of material characteristics. The complexation between blueberry leaf polysaccharide (BLP) and xanthan gum (XG) has not been reported, so we conducted this study.

The objective of this study is to develop a curcumin delivery system based on BLP-XG composite gel. Through comprehensive analysis, we characterized the structural characteristics, thermal stability, and rheological properties of the composite gels and explored the effects of BLP concentration on curcumin encapsulation efficiency, simulated gastrointestinal sustained release, and ultraviolet stability. The results of this study provide the theoretical foundation for effectively protecting and delivering curcumin while promoting the efficient use of blueberry leaf resources.

## 2. Materials and Methods

### 2.1. Materials and Reagents

Follow-up experiments were performed based on the BLP extracted from previous studies, which had the following composition: 61.53% ± 0.97% total sugars, 2.30% ± 1.52% proteins, and 18.37% ± 2.11% glucuronides [17]. Xanthan gum was sourced from Shandong Yousuo Chemical Technology Co., Ltd. (Linyi, China). Curcumin was obtained from Sinopharm Chemical Reagents Co., Ltd. (Shanghai, China). Simulated gastric fluid (SGF) was primarily made up of sodium chloride, dilute acid, and pepsin and supplied by Fuzhou Phygene Biotechnology Co., Ltd. (Fuzhou, China). Simulated intestinal fluid (SIF) was provided by Shanghai Macklin Biotechnology Co., Ltd. (Shanghai, China). Dialysis bags (MWCO: 8–14 kDa) were supplied by Shanghai Yuan Ye Technology Co., Ltd. (Shanghai, China). All reagents are analytically pure.

### 2.2. Preparation of BLP-XG Composite Gel Loaded with Curcumin

The collected blueberry leaves were washed, dried, ground, and sieved (60-mesh) to obtain blueberry leaf powder. The powder was dissolved in deionized water, and the mixture was ultrasonicated for 1.5 h, followed by stirring at 80 °C for 4 h (SHJ-6, Jintan Honghua Instrument Factory, Jintan, China). Then, the mixture was allowed to stand at room temperature for 12 h, and the supernatant was collected by centrifugation (JW-2032, Jiawen Instrument Equipment Co., Ltd., Hefei, China). The supernatant was concentrated using a rotary evaporator (RE-5205, Yarong Biochemical Instrument Factory, Shanghai, China), precipitated with 95% ethanol overnight, and centrifuged to obtain crude blueberry leaf polysaccharides (BLPs). The lyophilized BLPs were dissolved in deionized water at a ratio of 1:100 (*w*/*v*), and 20% (*v*/*v*) hydrogen peroxide solution (30% H_2_O_2_) was added for decolorization, followed by lyophilization (Alpha 1-4 LSCplus, Bomeixing Instrument Co., Ltd., Beijing, China). The decolorized sample was redissolved in deionized water under stirring, and Sevag reagent (n-butanol/chloroform = 1:4, *v*/*v*) was added. The mixture was shaken in a water bath for 1 h and then centrifuged at 3500 r/min for 10 min to separate the upper layer. This step was repeated until no protein layer was observed. Finally, the purified blueberry leaf polysaccharides (BLP) were obtained through dialysis and lyophilization [17].

The BLP-XG composite gel was prepared by referring to the method of Hou et al. [8] with modifications. In summary, the BLP stock solution was serially diluted to generate a series of concentrations and subsequently mixed with the XG solution at a volume ratio of 1:1 (*v*/*v*) at room temperature. The mixed solutions contained 1.0% (*w*/*v*) XG, while the BLP solutions ranged in concentration from 0 to 2.5% (*w*/*v*). Following a thorough stirring of the mixture with the magnetic stirrer, the temperature was elevated to 80 °C for 30 min to prepare a BLP-XG composite gel. This composite gel was then stored at a temperature of 4 °C for 24 h. A segment of the composite gel sample was subjected to freeze-drying for subsequent analysis. The encapsulation of curcumin was based on the experiments of others and modified according to the experiments [18]. An alcoholic solution of curcumin at a concentration of 1 mg/mL was prepared by first dissolving curcumin powder in anhydrous ethanol and then assisting dissolution by ultrasound. Subsequently, the curcumin alcohol solution was added drop by drop to the prepared composite gel samples, stirred and mixed well in the ratio of 1:20 (*v*/*v*), and then stored at 4 °C for 24 h. The BLP-XG composite gel samples loaded with curcumin (BLPX-CUR) were finally obtained. In order to prevent the degradation of curcumin, it is imperative to protect the sample from exposure to light during the preparation and analysis phases.

### 2.3. Determining the Properties of the BLP-XG Composite Gel

The morphology of the composite gel was investigated using scanning electron microscopy (SU8010, Hitachi, Ltd., Tokyo, Japan). In a nutshell, 0.05 g of the freeze-dried samples was deposited on conductive resin and sputter-coated with 10 nm of gold, and photographs were captured at an accelerating voltage of 5 kV with a magnification of 100× [19].

The crystallization and molecular amorphous properties of the samples were analyzed using an X-ray diffractometer (TD-3500, Dandong Tongda Technology Co., Ltd., Dandong, China). Measurements were conducted at room temperature with voltages of 40 kV and 40 mA and a scanning rate of 5° to 60°/min, as outlined previously [20].

Fourier transform infrared spectroscopy (FT-IR) (Nicolet iS 50+ Contiuum, Thermo Fisher Scientific, Waltham, MA, USA) was utilized for investigating the interaction forces between BLP and XG in prepared composite gel. The scanning procedures were performed at a temperature of 25 °C, a spectral resolution of 4 cm^−1^, and a wavenumber range from 4000 to 400 cm^−1^. Additionally, the spectrum of air was used as the background and control. For the measurement of particle size (average hydrodynamic diameter, nm) and zeta potential of the BLP-XG composite gel (before gelation), the sample underwent a dilution of 1:50 using deionized water before measurement with a particle size analysis zeta potentiometer (ZS90, Malvern, UK). These analyses were also performed at 25 °C, with each sample analyzed three times. The thermal characteristic of the samples was evaluated via thermogravimetric analysis (TGA) (Q500, TA Instruments, New Castle, DE, USA) following the methodology outlined by Hui & Gao [21], with minor adjustments. The sample was then put into an alumina crucible and subjected to heating from 30 °C to 600 °C at a consistent rate of 10 °C/min, utilizing nitrogen as the purge gas.

The rheological properties of the composite gels were assessed by employing a rotational rheometer (DHR-1, TA Instruments, New Castle, DE, USA) equipped with a parallel plate (40 mm parallel plate, 0.5 mm gap). Temperature control was achieved by means of an integrated Peltier system, as mentioned earlier [22]. The samples were loaded as follows: The ungelatinized BLP-XG composite gel was spread evenly in the center of the lower parallel plate, and the upper plate was lowered slowly to a 0.5 mm gap at a rate of 5 μm/s. The excess sample that overflowed the edges was carefully trimmed, and silicone oil was applied around the periphery to prevent evaporation. Subsequent to the completion of the loading process, the samples were equilibrated at 25 °C for a period of 10 min, with the objective of eliminating shear stress. Thereafter, the samples were subjected to testing. The composite gel samples were then exposed to an angular frequency scanning test at a constant temperature of 25 °C, with the scanning range set to 0.1–100 rad/s. The strain value was maintained at 0.5%, a level determined by an amplitude scan (strain 0.1–1000%, frequency 1 Hz) to ensure that the material was in the linear viscoelastic region. The gelation and melting temperatures of composite gels were explored by a temperature scanning test. The prepared BLP-XG composite gels were initially subjected to a temperature of 25.0 °C for 12 h. The sample temperature was reduced from 85.0 °C to 25.0 °C, maintained at 25.0 °C for 600 s, and then increased from 25.0 °C to 85.0 °C at a rate of 10 °C/min.

Atomic force microscopy (AFM) was employed to investigate the molecular chain conformation of the samples at 25 °C (Dimension ICON, Bruker Corporation, Billerica, MA, USA), following the methodology described by [23]. We used pure water as the solvent to dilute the gel samples, and their nanostructures were characterized using AFM. A precise volume of 10 μL from each gel was swiftly transferred to freshly cut mica and matured at room temperature for over 24 h prior to imaging. The nanostructures of all samples were analyzed in ambient air using tapping mode.

### 2.4. Encapsulation of Curcumin by BLP-XG Composite Gel

An appropriate amount of gel sample loaded with curcumin was initially centrifuged to remove free curcumin adsorbed on the surface of the gel samples and broken by physical shear and ultrasound-assisted extraction to release the encapsulated curcumin at room temperature. Then the extract was centrifuged at 8000 r/min for 20 min, and the supernatant was collected. This procedure was repeated until the supernatant was colorless. After combining the supernatants from multiple extractions, the absorbance was measured directly at a wavelength of 425 nm (UV-1780, Shimadzu Instruments Co., Ltd., Suzhou, China). Anhydrous ethanol was utilized as a control sample. The concentration of curcumin in the samples was determined by constructing a curcumin standard curve. The encapsulation efficiency (EE, %) was calculated according to the following equation.

EE (%) = C V/C_0_ V_0_ × 100 (1)

where C is the curcumin concentration in the supernatant (mg/mL), C_0_ is the curcumin concentration in the gel (mg/mL), V is the volume of the supernatant, and V_0_ is the volume of the solvent used to prepare the gel.

### 2.5. Simulated Gastrointestinal Release

Equal volumes of curcumin alcohol solution (Free-CUR) were mixed with SGF or SIF to serve as the blank control. In the experimental group, freshly prepared BLPX-CUR samples (2 mL) were placed in dialysis bags (molecular weight = 10 kDa) and then immersed in 30 mL of simulated SGF release medium. The 30 mL of simulated SGF release medium was incubated at 37 °C for 2 h with gentle shaking. After 2 h, the dialysis bag was transferred to 30 mL of simulated SGF release medium. It was then transferred to 30 mL of simulated SIF release medium and incubated for an additional 4 h [24]. During the incubation, 1 mL of the release medium was taken at predetermined time points. At these time points, 1 mL of the release medium (SGF and SIF were taken at 15 min and 30 min, respectively) was measured for curcumin concentration. Fresh medium was added in equal amounts to keep the volume of the release medium constant. The concentration of released curcumin is calculated from the absorbance values measured by a UV–visible spectrophotometer. The cumulative release rate of curcumin was calculated using the following equation.

Cumulative release rate (%) = Released amount/Total amount × 100%(2)

### 2.6. UV-Light Protection of Curcumin

In total, 3 mL of the gel samples was placed in a Petri dish, and about 0.3 mL of curcumin was sampled after irradiation for 0.5, 1, 2, 3, 5, and 7 h in a 30 W and 254 nm ultraviolet environment, and the retention rate of curcumin at different times was determined. Its absorbance is determined with a UV spectrophotometer at 425 nm. The curcumin content is calculated according to the curcumin standard curve. The retention rate (Rt) of curcumin is calculated using the following equation.

Rt (%) = (C_t_/C_0_) × 100 (3)

where C_0_ represents the initial concentration of curcumin (μg/mL), and C_t_ represents the concentration of curcumin after ultraviolet treatment for a certain number of hours (μg/mL).

### 2.7. Statistical Analysis

All experiments were conducted in triplicate, and the results are presented as means with standard deviation. Data analysis and plotting using Excel and Origin 2024. Statistical significance was determined using one-way analysis of variance (ANOVA) followed by the LSD test, and *p* < 0.05 was considered a significant difference.

## 3. Results and Discussion

### 3.1. Particle Size and Potential Analysis

**Figure 1:**
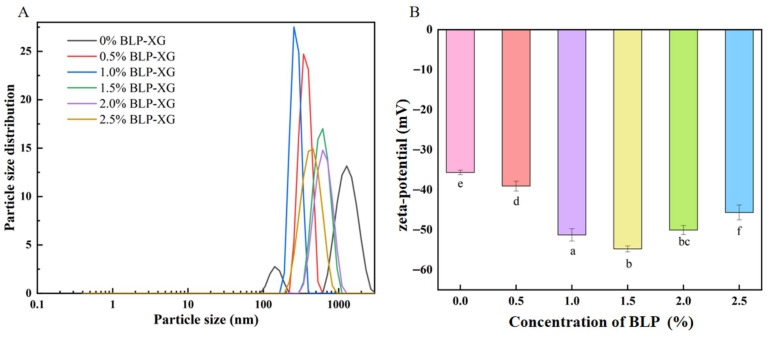
Particle size (**A**) and zeta potential (**B**) of BLP-XG composite gel at different BLP concentrations. Values with distinct letters (a–f) indicate significant differences (*p* < 0.05).

The particle size and zeta potential characteristics of BLP-XG composite gel are shown in Figure 1. The measured particle size and zeta potential refer to the macromolecular aggregates that form the gel, rather than the gel itself. Studies have generally examined the particle size of samples as an indicator of their aggregation, which affects the gelation characteristics of the composite gel. The structural stability of gels exhibits a positive correlation with reduced particle size, as smaller particulate dimensions promote more homogeneous network formation and enhanced intermolecular interactions [25]. The average particle size of BLP-XG composite gels demonstrated a biphasic response to increasing polysaccharide concentration, initially decreasing before exhibiting a subsequent increase (Table 1). This non-monotonic behavior suggests competing mechanisms of molecular interaction and aggregation at different concentration ranges (Figure 1A). Pure XG exhibited the largest mean particle diameter among all tested samples. Notably, the incorporation of BLP resulted in a significant reduction for the BLP-XG composite gel. The particle size of the composite gel reached its minimum value of 275.6 nm when the BLP concentration was 1.0%. This pronounced decrease suggests that BLP facilitates the formation of a more homogeneous and thermodynamically stable network structure through enhanced intermolecular interactions between the polysaccharide components.

Zeta potential serves as a crucial indicator of the attractive and repulsive forces between macromolecules within the gel. A higher absolute value of zeta potential denotes enhanced stability of the gel system [26]. BLP is an acidic polysaccharide that typically carries a negative charge, with charge density increasing as the concentration rises. As the proportion of polysaccharide increases, the overall electronegativity of the composite also increases. At lower polysaccharide concentrations, the zeta potential of the composite gel is primarily influenced by XG. When the polysaccharide content is at a moderate level, the potential value of the composite continues to increase, although the rate of increase starts to slow down due to charge neutralization effects. As the polysaccharide concentration continues to rise, the charge neutralization effect becomes more pronounced, leading to a slight decrease in the zeta potential value (Figure 1B).

### 3.2. FT-IR Analysis

**Figure 2:**
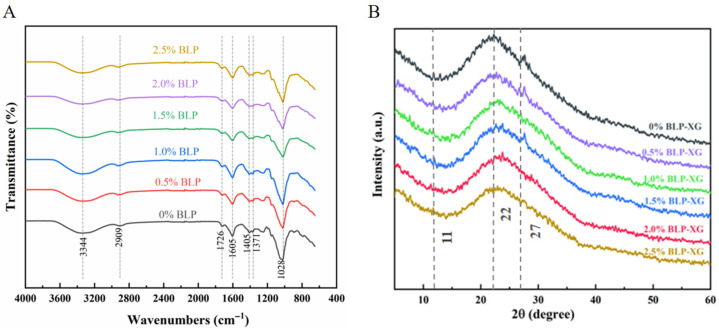
FT-IR (**A**) and XRD (**B**) images illustrating the BLP-XG composite gels.

FT-IR spectroscopy serves as an effective analytical method for identifying functional groups and characterizing chemical bond modifications in polysaccharide samples. FT-IR analysis revealed characteristic absorption bands corresponding to amide A (~3300 cm^−1^) and amide I (1600–1700 cm^−1^) in all gel samples. While these spectral features were consistently present across samples, quantitative analysis demonstrated measurable variations in peak intensities at specific wavenumbers (Table 2), indicating subtle differences in molecular conformation and intermolecular interactions (Figure 2A). Notably, no new peaks were observed following the BLP-XG, suggesting that the interactions within the samples were non-covalent in nature. The wider absorption peak observed at 3344 cm^−1^ corresponds to the O-H and hydrogen bond stretching vibrations, indicating a shift in the composite gel’s peak to a lower wavenumber. This shift likely resulted from the intricate vibrational stretching of inter- and intramolecular –OH groups, indicating the presence of hydrogen bond interactions in the gel [27]. The peak around 2909 cm^−1^ corresponded to the C–H stretching vibration of hydroxyl alcohol [28], which corresponds to the absorption peak of polysaccharides. The distinct absorption peak observed at 1726 cm^−1^ corresponds to the characteristic C=O stretching vibration of acetyl functional groups. This spectroscopic signature is particularly significant, as acetylation plays a crucial role in the gelation process, influencing both intermolecular crosslinking and network stability through ester bond formation and hydrogen bonding interactions [29]. The peak at 1605 cm^−1^ corresponds to the asymmetric stretching of –COO, indicating the presence of glucuronic acid, consistent with the chemical composition of BLP. The characteristic absorption peaks between 1560 and 1335 cm^−1^ reflect the N–H stretching vibrations associated with proteins. The “fingerprint” region for carbohydrates, ranging from 800 to 1200 cm^−1^, aids in identifying the principal chemical groups within the polysaccharides, while the absorption peak at 1025 cm^−1^ is linked to the C–O–C stretching of the pyran ring.

After observing the amide I and II bands, the characteristic peak intensity of the mixture continuously decreased after the concentration of BLP gradually increased, which was caused by electrostatic and hydrophobic interaction between BLP and XG [30]. Consequently, the synergistic interactions between XG and varying concentrations of BLP primarily derive from hydrogen bonding, as well as electrostatic and hydrophobic forces. These findings further suggest that the addition of BLP modified the gel characteristics of XG.

### 3.3. Scanning Electron Microscopy Analysis of BLP and Composite Gel

**Figure 3:**
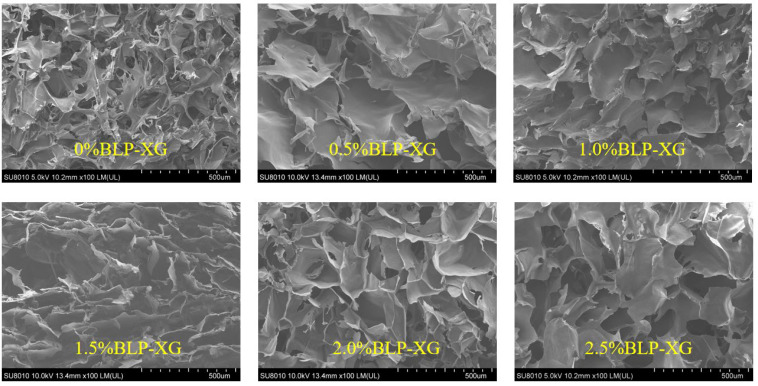
Influence of various BLP additions on the SEM pictures of BLP-XG composite gels.

Scanning electron microscopy is an effective method for visualizing the internal structure of gels. The microscopic morphology of the composite gels with different BLP additions showed that the gel with no BLP or containing XG alone had a rough surface with broken overall morphology and irregular pores, while the gel with the added BLP exhibited a smooth surface with continuous pores (Figure 3). When 1.0% of BLP was added, the pores of the composite gel were uniform, dense, and orderly but were fragmentized as the BLP concentration increased, indicating that the high charge of XG itself could have resulted in the electrostatic repulsion. Based on previous results of FT-IR, it can be inferred that XG and BLP acted together through hydrogen bonds and electrostatic effects, while the polysaccharide could have served as a bridge between different molecular chains of XG to reduce the distance between different molecular chains and form a compact three-dimensional network structure [31].

### 3.4. Physical and Chemical Properties of the BLP-XG Composite Gel

The crystal structure of the sample can be obtained non-destructively by XRD. Figure 2B presents the XRD pattern of BLP-XG containing varying concentrations of BLP. The main diffraction peak of the BLP-XG composite gel was identified at 2θ = 22°, indicating that the gel predominantly presents semi-crystalline and amorphous characteristics. In addition, two distinct sharp peaks were detected at 2θ values of 11° and 27°, implying that crystalline domains are present within the polymer. However, with increasing concentrations of BLP, the diffraction peak at 2θ = 27° diminished and ultimately disappeared. This observation implies that the structural adjustments occurring during gel formation may inhibit the manifestation of polysaccharide micro-crystals [32].

**Figure 4:**
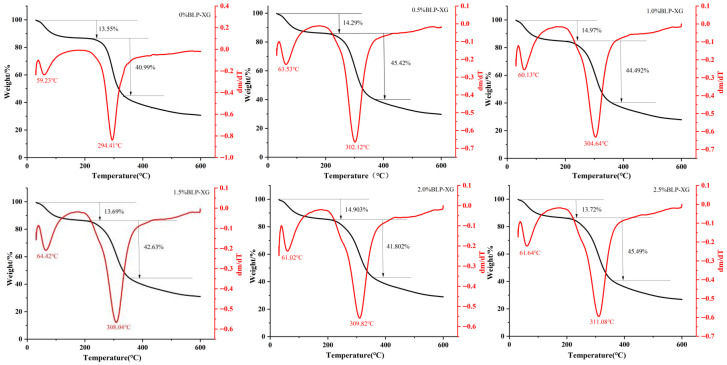
Thermal stability of BLP-XG composite gels. TGA curves (black line) and DTG curves (red line).

Since thermal stability significantly influences the functionality and performance of gel-based products, we employed TGA to evaluate the thermal stability of the composite gel as illustrated in Figure 4. The TGA curve depicts the residual mass of the decomposed sample as a function of increasing temperature [33]. The first-order derivative of the TGA curve is generally represented by the DTG curve, expressing the rate of mass alteration during heating [34]. Based on the DTG curve, the sample mass decreases with rising temperature, consistent with the thermal decomposition behavior of the material at varying temperature ranges. In this study, the thermal decomposition of BLP-XG composite gel occurred in three distinct stages. The first stage (20–150 °C) exhibited a mass loss of approximately 15%. This primarily involved the evaporation of polysaccharide-bound water and volatile components, as well as the removal of water adsorbed by hydrophilic groups in the gel matrix. The second stage (150–450 °C) exhibited a mass loss of approximately 45%, likely due to the degradation of polysaccharides and the breakdown of weaker chemical bonds. The third stage occurs at 450–600 °C with less mass loss than the second stage, but the weight loss rate of the BLP-XG composite gel is relatively high. For example, when the content of BLP-XG was 2.5%, the residual amount was 27.43%, indicating the lower weight loss and improved thermal stability of the composite gel compared to that of pure XG. This thermal behavior may be associated with the breakage of polysaccharide chains and alterations in the gel network structure at elevated temperatures. Further verification of changes in hydrogen bonds and intermolecular interactions may be necessary using spectroscopic techniques [35].

### 3.5. Rheological Analysis

**Figure 5:**
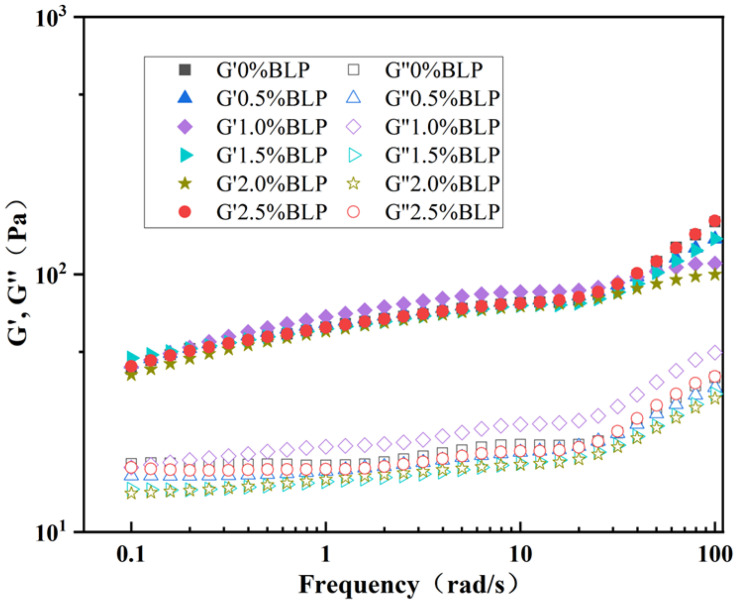
The analysis of the dynamic rheological behavior of BLP-XG composite gels.

To study the viscoelastic behavior of the composite gel system, dynamic frequency scanning was used. For elastic behavior, G′ > G″ and G′ < G″ are characteristic of viscous behavior. The results demonstrated that within the complete frequency range, the storage modulus of all composite gels significantly exceeded the loss modulus (G′ > G″), with no obvious frequency dependence, indicating the composite gels had a solid-like structure with dominant elasticity (Figure 5). This reflected excellent storage stability, as the gels could resist deformation and maintain structural integrity during long-term storage. All mixtures exhibited a cohesive network structure and robust resilience under mechanical stress [36]. The results suggested that although BLP incorporation did not significantly alter the viscoelastic response pattern, it could affect the gel’s mechanical strength at specific concentrations. For instance, at a BLP concentration of 1.0%, the storage modulus of the gel was at the maximum (85.45 Pa at 10 rad/s), which was significantly higher than that of the other concentration groups (*p* < 0.05), likely due to the enhanced structural stability conferred by the optimized polysaccharide concentration. Compared to XG alone, BLP-containing gels displayed higher stability, which is consistent with the previous thermal stability results. However, beyond this concentration, the storage modulus decreased, possibly attributed to the interference with the hydrogen bonds and various non-covalent interactions among gel molecules, as well as weakened intermolecular forces within the polymer chains. These findings further indicated that the composite gel possessed a stable gel network with long-term storage stability.

**Figure 6:**
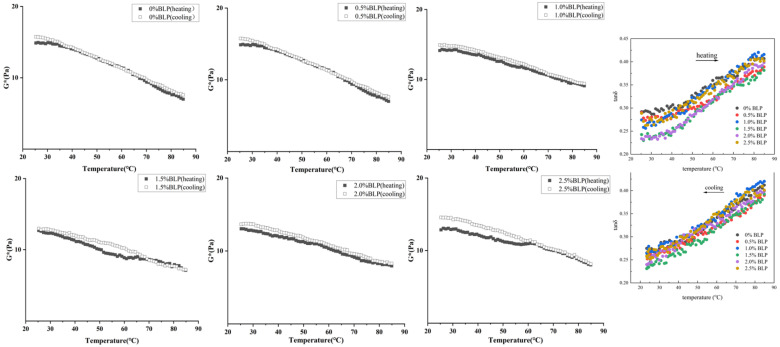
Complex viscosity diagrams of BLP-XG composite gels with a maximum error of ±0.5 Pa.

Temperature scanning reveals the transition behavior from gel to sol states. The intersection of G′ and G″ was a common indicator of the sol–gel transition temperature (T_gel_), but the criteria for determining T_gel_ varied across different material systems. In this study, there was no distinct intersection point within the test temperature range, G′ and G″ of BLP and XG composite gel, and no intuitive T_gel_ and T_melt_ of the gel (Figure 6). Consequently, the composite modulus G* was introduced, with the T_melt_ defined as the onset temperature where G* begins to decrease during the instrument-controlled cooling ramp. G* of BLP-XG composite gel decreased progressively with increasing temperature during the heating process. The molecular structure of native XG primarily consisted of helical configurations with supplementary disordered chains. As the temperature increased, the thermal energy initiated the rupture of intermolecular hydrogen bonds within the gel. Concurrently, other intermolecular forces, including van der Waals forces and hydrophobic interactions, were progressively disrupted. The XG helical structure, which had been originally maintained by these forces, lost its support, and the ordered helical configuration gradually uncoiled into a disordered random coil [37]. Concurrently, the cross-linking effect of the molecular chains in the gel network gradually weakened, causing the molecular chains to relax from their tightly entangled state. The modulus of the BLP-XG composite gel during heating and cooling was highly coincident, and the transition temperature was difficult to distinguish. The loss tangent (tanδ = G″/G′) provides additional insight into the viscoelastic behavior of the gels. All samples exhibited tanδ values below 1 throughout the temperature range, confirming their gel-like character. The tanδ profiles showed no distinct peak corresponding to gel–sol transition, further supporting our conclusion that no clear transition temperature could be identified within the tested range. This phenomenon may have been related to the mild unwinding and recovery process. Since the spiral cannot be unwound onto a disordered chain, the change in modulus during the thermal transition was not obvious. Notably, the gel-to-sol conversion temperature could not be determined for the composite gel, potentially due to the specific XG variety used in this study, which is consistent with previous findings [38].

### 3.6. Morphology of the Composite Gel

**Figure 7:**
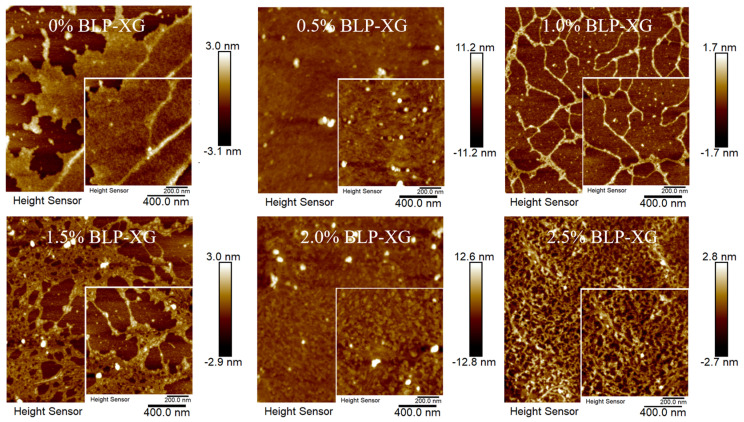
Nanostructure of BLP-XG mixtures with different BLP contents.

AFM was employed to characterize the morphology features of composite gel samples containing varying polysaccharide concentrations. The microstructure analysis revealed significant alterations with the addition of BLP (Figure 7). At 0.5% BLP concentration, the composite gel formation was initiated through molecular entanglement between BLP and XG, resulting in a homogeneous, dense polymeric network with enhanced structural stability. When the BLP concentration reached 1.0%, the XG chain showed a spiral structure and further aggregated into a three-dimensional network structure. However, with the increase in polysaccharide concentration, the network structure of the composite gel was more closely cross-linked. The average surface roughness of the composite gels with 0–2.5 wt% of BLP was 0.558 nm, 0.881 nm, 0.283 nm, 0.530 nm, 1.16 nm, and 0.600 nm, respectively. These results substantiate that BLP could form a more stable network structure at an appropriate concentration [39].

### 3.7. Analysis of Curcumin Encapsulation Efficiency

**Figure 8:**
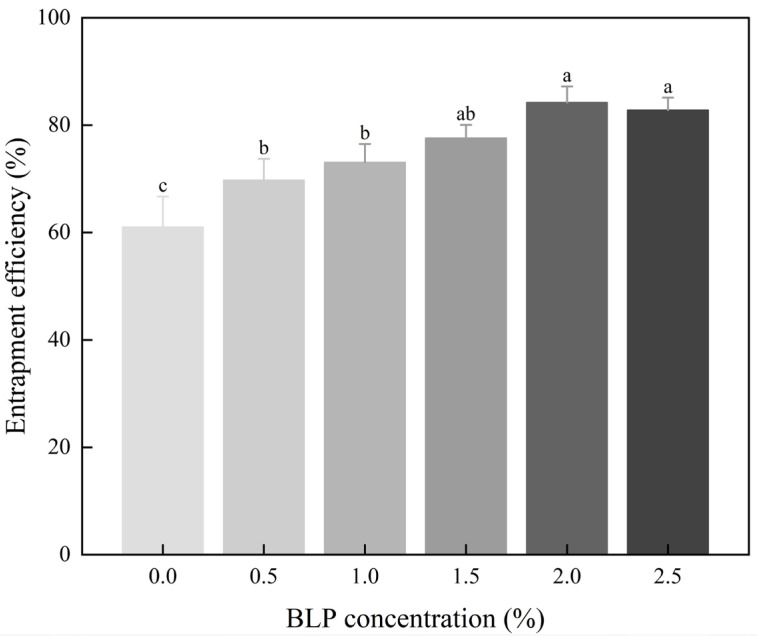
The encapsulation efficiency of curcumin in the BLP-XG composite gel. Groups labeled with different symbols differ significantly (*p* < 0.05); identical letters indicate no significant difference.

The overall EE of the BLP-XG composite gel system was higher than that of curcumin encapsulated by pure XG (Figure 8). EE exhibited an initial increase, followed by a subsequent decrease, in response to the gradual increase in BLP concentration, which ranged from 0 to 2.5% (*w*/*v*). The EE reached a maximum of 84.23% at a BLP concentration of 2.0%, indicating that the BLP addition altered the gel properties of XG and enhanced gel stability. EE entered a plateau phase at BLP concentrations of 1.5–2.0%, followed by a decrease at 2.5%. Although this decrease was not statistically significant, the results indicated the presence of an optimal BLP concentration window. Beyond this threshold, excessive viscosity changes might have occurred, potentially altering the gel structure. However, this effect would have required verification with a larger sample size.

### 3.8. Study of Simulated Gastrointestinal Controlled Release

**Figure 9:**
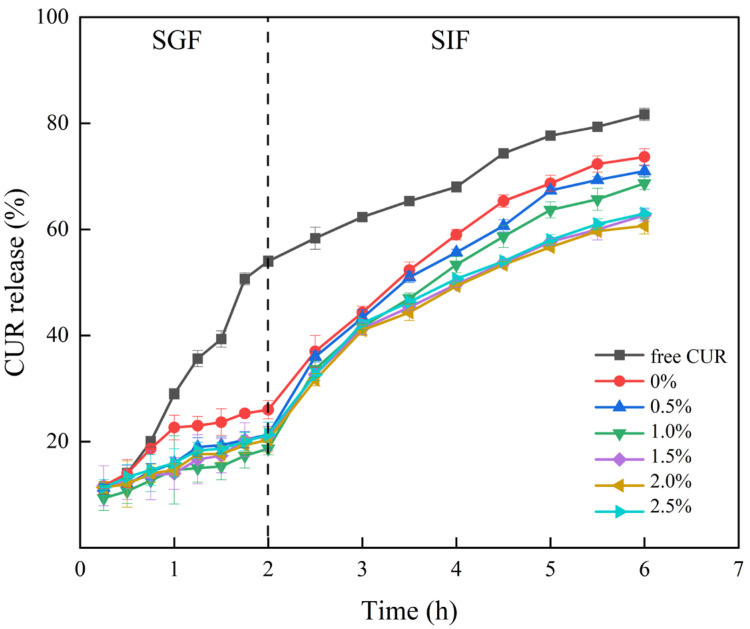
Release rates of BLPX-CUR composite gels at different BLP concentrations after different periods of time in SGF and SIF.

The cumulative release profiles of BLPX-CUR complexes in SGF and SIF were illustrated in Figure 9. The overall release rate of free curcumin was higher than that of the BLP-XG composite gel. The chemical stability of curcumin was closely related to pH, with higher stability under acidic conditions (e.g., SGF) than under alkaline conditions (e.g., SIF). The BLPX-CUR complexes demonstrated a slower release trend than free CUR in SGF, with a curcumin release rate of less than 23% after two hours of simulated gastric fluid digestion. After transfer to SIF, a relatively high release rate was observed during the initial hour of release, followed by a leveling off of the release rate during the subsequent period. The BLP-XG composite gel protected curcumin from the acidic environment of the stomach to some extent while promoting the sustained release of the active ingredient in the small intestine. This two-phase release characteristic suggested that BLP-XG composite gel had better sustained release properties than pure XG. These findings could help in the development of a delivery system that effectively retained and protected bioactive substances in the stomach while promoting their release in the small intestine, thereby improving bioavailability.

### 3.9. Ultraviolet Irradiation

**Figure 10:**
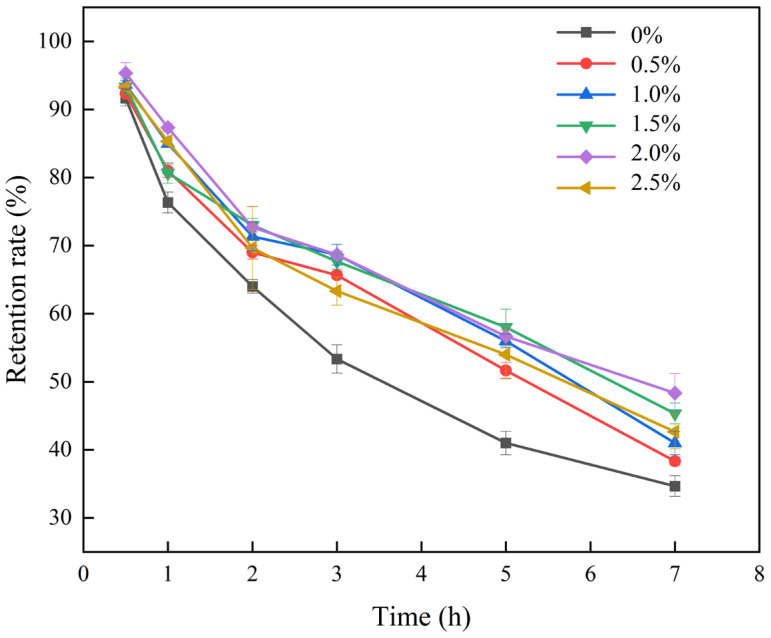
Retention rates of BLPX-CUR composite gels at different BLP concentrations after different UV irradiation times.

Ultraviolet (UV) irradiation is a widely employed sterilization method for food and medicine, but it was employed to simulate accelerated light exposure to evaluate the efficacy of the gel in protecting curcumin from light-induced degradation in this study. Curcumin is susceptible to UV radiation due to its polyphenolic nature and is prone to UV-induced degradation [40]. Therefore, the retention of curcumin following UV radiation was employed as an indicator of the protective effect of CUR stabilized by BLP-XG composite gels. In the case of pure XG, curcumin exhibited the most rapid degradation rate when exposed to UV irradiation. This phenomenon was attributed to the exposure of the curcumin molecule to UV light, which promoted the degradation reaction in an inverse manner. In the BLP-XG composite gel system, the interfacial layer of the BLP-XG composite gel functioned as a barrier to UV radiation, thereby restricting the degradation reaction at the interface and preserving the integrity of the curcumin molecule. As demonstrated in Figure 10, following a 7 h UV irradiation period, the BLP-XG composite gel exhibited enhanced resistance to UV irradiation. These findings suggest that the incorporation of BLP into the gel may have augmented its protective effect on curcumin.

## 4. Conclusions

The novel BLP-XG composite gels were successfully prepared, and the addition of BLP resulted in a significant increase in the thermal decomposition temperature of XG. It was observed that the BLP-XG composite gel had a smaller particle size and more homogeneous structure at a polysaccharide concentration of 1.0%. The composite gel exhibited solid-like properties, and BLP was attached to XG in a molecular chain conformation. The composite gel’s stable structure was attributed to the interplay of hydrogen bonding and electrostatic interactions. The encapsulation effect of the BLP-XG composite gel on curcumin was noteworthy, as it facilitated the compound’s retention in gastric juice and subsequent entry into the intestine. BLP-XG demonstrated a better protective effect against curcumin under UV irradiation. These findings suggested avenues for the implementation of the BLP-XG composite gel system and the enhancement of the application scope of other additives, while fostering the comprehensive development of blueberry leaf extract.

## Figures and Tables

**Table 1:** Mean diameter and polydispersity index (PDI) of BLP-XG composite gels with different BLP concentrations.

BLP Concentration	D_n_ (nm)	D_v_ (nm)	PDI
0%	964.7 ± 11.2	1398.7 ± 18.5	0.451 ± 0.004
0.5%	395.8 ± 6.0	437.6 ± 5.9	0.107 ± 0.002
1.0%	259.7 ± 3.4	275.6 ± 3.9	0.059 ± 0.001
1.5%	552.3 ± 5.4	601.5 ± 6.8	0.089 ± 0.001
2.0%	578.6 ± 6.8	622.4 ± 7.2	0.075 ± 0.001
2.5%	429.8 ± 5.8	464.5 ± 6.5	0.081 ± 0.001

Note: Values are mean ± standard deviation (*n* = 3).

**Table 2:** Fourier transform infrared spectral transmittance of composite gels with different BLP concentrations at characteristic wavenumbers.

Wavenumbers (cm^−1^)	Transmittance (%)					
	0% BLP	0.5% BLP	1.0% BLP	1.5% BLP	2.0% BLP	2.5% BLP
3344	85.31	85.98	83.46	89.25	90.62	86.71
2909	92.87	92.70	92.19	94.56	94.92	93.11
1726	92.51	92.33	91.09	93.78	93.78	91.84
1605	82.24	82.85	79.77	86.31	86.31	81.87
1405	85.82	85.72	83.22	88.16	88.08	84.42
1371	86.29	85.93	83.52	88.35	88.27	84.66
1028	57.65	58.67	52.66	69.90	65.65	57.35

## Data Availability

The original contributions presented in the study are included in the article; further inquiries can be directed to the corresponding author.
